# Comparative Study Reveals Insights of Sheepgrass (*Leymus chinensis*) Coping With Phosphate-Deprived Stress Condition

**DOI:** 10.3389/fpls.2019.00170

**Published:** 2019-02-19

**Authors:** Lingyu Li, Haomeng Yang, Lianwei Peng, Weibo Ren, Jirui Gong, Peng Liu, Xinhong Wu, Fang Huang

**Affiliations:** ^1^Key Laboratory of Photobiology, Institute of Botany, Chinese Academy of Sciences, Beijing, China; ^2^University of Chinese Academy of Sciences, Beijing, China; ^3^Shanghai Key Laboratory of Plant Molecular Sciences, College of Life Sciences, Shanghai Normal University, Shanghai, China; ^4^Institute of Grassland Research, Chinese Academy of Agricultural Sciences, Hohhot, China; ^5^College of Resources Science and Technology, Beijing Normal University, Beijing, China

**Keywords:** *L. chinensis*, Pi-deprivation, low-Pi stress, ATP synthase, photosynthesis

## Abstract

Sheepgrass [*Leymus chinensis* (Trin.) Tzvel] is a valuable forage plant highly significant to the grassland productivity of Euro-Asia steppes. Growth of above-ground tissues of *L. chinensis* is the major component contributing to the grass yield. Although it is generally known that this species is sensitive to ecosystem disturbance and adverse environments, detailed information of how *L. chinensis* coping with various nutrient deficiency especially phosphate deprivation (-Pi) is still limited. Here, we investigated impact of Pi-deprivation on shoot growth and biomass accumulation as well as photosynthetic properties of *L. chinensis*. Growth inhibition of Pi-deprived seedlings was most obvious and reduction of biomass accumulation and net photosynthetic rate (Pn) was 55.3 and 63.3%, respectively, compared to the control plants grown under Pi-repleted condition. Also, we compared these characters with seedlings subjected to low-Pi stress condition. Pi-deprivation caused 18.5 and 12.3% more reduction of biomass and Pn relative to low-Pi-stressed seedlings, respectively. Further analysis of *in vivo* chlorophyll fluorescence and thylakoid membrane protein complexes using 2D-BN/SDS-PAGE combined with immunoblot detection demonstrated that among the measured photosynthetic parameters, decrease of ATP synthase activity was most pronounced in Pi-deprived plants. Together with less extent of lipid peroxidation of the thylakoid membranes and increased ROS scavenger enzyme activities in the leaves of Pi-deprived seedlings, we suggest that the decreased activity of ATP synthase in their thylakoids is the major cause of the greater reduction of photosynthetic efficiency than that of low-Pi stressed plants, leading to the least shoot growth and biomass production in *L. chinensis*.

## Introduction

Phosphorus (P) is a major nutrient essential to plant growth and development. It is an intrinsic component of ATP, phospholipids as well as nucleic acids and plays a central role in plant cell metabolism. Though P is abundant on Earth, inorganic phosphate anion (Pi), the bioactive form for plants, is frequently limited in soils due to its low availability (Schachtman et al., 1998; Raghothama and Karthikeyan, 2005). It is estimated that that 30% of the agricultural soils worldwide are Pi deficient and concentration of Pi in soil solutions is generally lower than 10 μM, which is far below the critical level required for the optimal performance of crops, resulting in severe yield limitations (Batjes, 1997; Abel et al., 2002; MacDonald et al., 2011). On the other hand, over-addition of Pi exists in some other regions, causing eutrophication of lakes and seas (MacDonald et al., 2011) as well as agricultural run-off (Abel et al., 2002; Niu et al., 2013). Therefore, it is imperative to increase Pi utilization efficiency of plants toward sustaining global vegetation productivity with reduced Pi fertilizers (López-Arredondo et al., 2014).

Sheepgrass *Leymus chinensis* (Trin.) Tzvel (hereafter *L. chinensis*) is perennial forage plant significant to Euro-Asia grassland productivity. The species is dominant in the steppes and its growth status is fundamental to the grassland ecosystem as well as the regional forage yields (Bai et al., 2004; Liu et al., 2008; He et al., 2017). Compared to other forage species, however, *L. chinensis* is more sensitive to ecosystem disturbance and stress conditions including water deficiency (Bai et al., 2004; Xu and Zhou, 2006). Therefore, it has been used as a popular model grass system for ecological as well as physiological research (Chen et al., 2013; Li et al., 2015, 2016; He et al., 2017). Based on the large-scale survey, leafy P content of land plants distributed in China is considerably lower than the global averages that excluded Chinese species. This suggests that Pi deficiency is common in most soils of China (Han et al., 2005) and substantial effort should be made toward increasing efficiency of Pi utilization in diverse plant species. To fulfill this, detailed information of valuable plants, such as *L. chinensis*, to Pi-deficiency tolerance as well as their acclimation responses/mechanisms is required.

During the past decades, numerous studies have been conducted to investigate plant responses to Pi-deprivation mainly using the model systems such as Arabidopsis and rice. In general, Pi-deprivation leads to rapid inhibition of shoot growth as well as changes in various metabolic processes (Chiou and Lin, 2011; Veneklaas et al., 2012). Recent advances in Pi-stress biology have allowed identification of several key proteins, such as PHR1 (Phosphate Starvation Response 1) and PHT1 (Phosphate Transporter 1), involved in Pi sensing or/and homeostasis. Transcriptomic and proteomic analyses in Arabidopsis also revealed a large number of differentially expressed genes/proteins, suggesting that reprogramming of metabolic and regulatory networks occurs subjected to Pi-deprivation (Hammond et al., 2003; Wu et al., 2003; Misson et al., 2005; Morcuende et al., 2007). Nevertheless, cross comparison of the results from the different experiments revealed little agreement and these discrepancies are of indications that the Pi-starvation response, even in the same plant species, is highly dynamic and influenced by the plant age and growth conditions (Morcuende et al., 2007; Alexova and Millar, 2013). Apart from the research in Arabidopsis, physiological studies of several crops such as rice and maize have also revealed that traits for high phosphate use efficiency are rarely conserved between species (Li et al., 2007, 2010; Torabi et al., 2009; Yao et al., 2011; Alexova and Millar, 2013). Thus, studying the physiology of Pi-deprivation of economically important plant species, such as the valuable grass *L. chinensis*, is critical to rationalize the Pi-supply in the natural and cultivation systems.

Above-ground tissue biomass derived from photosynthesis is the major part contributing to grass productivity of *L. chinensis*. To understand how Pi starvation affects its above-ground tissue growth, we have initially investigated low-Pi effect on its photosynthetic properties and revealed for the first time a close correlation of photosystem II (PSII) accumulation and external Pi concentration in the medium (Li et al., 2016). The questions arise how Pi-deprivation affects above-ground tissue growth of *L. chinensis* and whether the correlation exists under Pi-deprived stress condition.

In the present work, we have investigated impact of Pi-deprivation on shoot growth, leafy biomass accumulation and photosynthetic properties of *L. chinensis*. We also compared these characters quantitatively with that subjected to low-Pi stress treatment. We show that Pi-deprivation led to remarkable reduction of shoot growth, biomass accumulation as well as net photosynthesis rate (Pn) in the leaves. We found that among the measured photosynthetic parameters the decrease of ATP synthase activity in Pi-deprived plants was significantly greater than that under low-Pi stress condition. We address that the greater inhibition of photosynthetic efficiency under Pi-deprivation is mainly attributed to down-regulated ATP synthase activity in thylakoids of *L. chinensis*.

## Materials and Methods

### Plant Material, Growth Conditions, and Pi-Deprivation Treatment

Seeds of *L. chinensis* (Trin.) Tzvel were germinated as described (Li et al., 2016). Two-week-old seedlings were transplanted to a vessel (350 mL) containing 1/4 strength Hoagland solution (Hoagland and Arnon, 1950) and cultured for an additional 4 weeks in a growth chamber with a light: dark cycle of 14:10 h (25:20°C) and light intensity and humidity was controlled at 280 μmol/m^2^ s and 50%, respectively. The solution was aerated via air bubbling and changed once a week. For stress treatment experiments, roots of the 4-week-old seedlings were carefully washed with fresh medium either containing repleted Pi (250 μM, control), low-Pi (2.5 μM) as described in the previous work (Li et al., 2016) or deprived-Pi (0 μM) and grown for additional 4 weeks in the controlled environment described above. In the low-Pi and deprived-Pi solutions, KH_2_PO_4_ were substituted by an equimolar amount of K_2_SO_4_ according to Wu et al. (2003) and the solutions were replenished every 2 days. Three replicates each consisting of five seedlings were included for both control and Pi-stress treatments. For biomass determination, shoot from individual plants of each treatment was excised and weighed after oven dried. For other measurements, roots and the third leaves of the corresponding seedlings were either sampled for fresh use or frozen in liquid nitrogen then stored at -80°C.

### Quantification of Pi and Anthocyanin Contents

Inorganic phosphate (Pi) content in the root and leaf tissues was determined as described (Zhou et al., 2008; Li et al., 2016). Anthocyanin was quantified according to (Wang et al., 2011). Frozen leafy tissue (100 mg) from each treatment was used and relative amount of anthocyanin was calculated as A530/g fresh weight.

### Chlorophyll Content and Chlorophyll a Fluorescence Analysis

Chlorophyll content was measured using the method (Arnon, 1949). Chlorophyll *a* fluorescence was measured with a chlorophyll fluorimeter (Dual PAM-100) (Heinz Walz, Effeltrich, Germany) by following the manufacturer’s instructions. Fo and Fm represent the minimal and maximal fluorescence in the dark-adapted state (30 min), respectively. F is the steady-state level of fluorescence emission. Fm’ is the maximal fluorescence of the light-adapted state. The maximal and effective photochemical quantum yield of PSII was calculated as Fv/Fm = (Fm-Fo)/Fm and φ(II) = (Fm’-F)/Fm’, respectively. Non-photochemical quenching (NPQ) was calculated as (Fm-Fm’)/Fm’. Light-induced redox changes of P_700_ was measured as previously (Yang et al., 2014; Xing et al., 2017) using the PAM101 fluorimeter (Heinz Walz, Effeltrich, Germany) equipped with a dual-wavelength P_700_ unit (ED800T). Absorbance changes at 820 nm induced by saturating far-red light illumination (720 nm, 24 μmol/m^2^ s) represent the relative amounts of photoxidizable P_700_ (Klughammer and Schreiber, 1998).

### Thylakoid Membrane Preparation, 2D BN-PAGE, and Immunoblot Analysis

Chloroplasts isolation and thylakoid membrane preparation were performed as described (Munekage et al., 2002; Peng et al., 2008). Isolated chloroplasts were osmotically ruptured in a buffer containing 20 mM HEPES/KOH (pH 7.6), 5 mM MgCl_2_, and 2.5 mM EDTA. Thylakoid membranes were then collected by centrifugation and resuspended in the same buffer. The resulting thylakoid membranes were used freshly for 2D BN-PAGE as described (Yang et al., 2014; Zhao et al., 2017) with modifications according to (Peng et al., 2008). Briefly, thylakoid membranes (equivalent to 10 and 5 μg chlorophyll for Coomassie staining and immunoblot detection, respectively) were solubilized with 1% n-dodecyl-β-D-maltoside then subjected to BN-PAGE (5–12% acrylamide, 4°C) with the running program of 50 V for 30 min followed by increasing voltage gradually up to 200 V. For protein separation in the second dimension, the lanes of BN gel equilibrated in the SDS sample buffer containing 5% β-mercaptoethanol and 8 M urea were placed on the top of 15% SDS-PAGE gel (Laemmli, 1970) and the proteins was separated by electrophoresis with AE-6500 apparatus (ATTO, Japan). Proteins were detected by Coomassie Brilliant Blue R-250 staining or by immunoblotting using antibodies against photosynthetic proteins purchased from Agrisera (Umeå, Sweden). The dilution for the specific antibodies is as follows: anti-D1 (1:4,000); anti-CP43 (1:3,000); anti-PsaA (1:5,000); anti-PsaB (1:3,000). The gels were scanned using a UMAX Power-Look 2100XL scanner (Willich, Germany) and the immuno-signals were visualized by a Chemiluminescent Imaging System (LuminoGraph WSE-6100; ATTO, Japan). Protein content was determined according to Peterson (1977) using BSA as standard.

### Determination of MDA Content and Enzyme Activity

Malondialdehyde (MDA) content determination and enzyme activity assay were done as previously (Li et al., 2016). For MDA content, liquid N_2_-frozen leaves (100 mg) were homogenized with 5% (w/v) trichloroacetic acid (TCA) then centrifuged at 3,000 *g* for 15 min. For the assay, the supernatant (0.2 ml) was mixed with 0.5 ml of 0.5% TBA solution and boiled for 10 min. The reaction was stopped on ice followed by centrifugation (3,000 *g*) for 15 min. The absorbance of the resulting supernatant at 450, 532, and 600 nm was determined vs. a blank in which TBA was omitted to obtain the correct MDA concentration (Hodges et al., 1999). For enzyme activity assay, crude extracts were prepared by grinding the liquid N_2_-frozen samples (100 mg) and extracted in 3 ml of 50 mM potassium phosphate buffer (pH 7.8) containing 0.2 mM EDTA and 1% (w/v) polyvinylpyrrolidone (PVP). After centrifugation for 20 min (10,000 *g*, 4°C), the supernatant was saved and used for respective enzyme activity measurement. Peroxidase (POD, EC 1.11.1.7) and superoxide dismutase (SOD, EC 1.15.1.1) activity was determined according to the methods (Trevisan et al., 1997; Caverzan et al., 2014). For POD, one enzyme unit was defined as per 0.1 increase or decrease of absorbance at 470 nm per min. One enzyme unit for SOD was defined as the amount of enzyme extract corresponding to 50% inhibition of the reaction (Beauchamp and Fridovich, 1971). Enzymatic activity was expressed as enzyme units per miligram protein.

### Estimation of ATP Synthase Activity

ATP synthase activity was estimated based on H^+^ conductivity (gH^+^) through ATP synthase using the Dual PAM-100 instrument equipped with a P515/535 module (Heinz Walz, Effeltrich, Germany) (Schreiber and Klughammer, 2008). Measurements were performed by following to the manufacturer’s instructions and as described previously (Duan et al., 2016; Zhang et al., 2018). Briefly, seedlings were dark-adapted overnight before illumination with actinic light (754 μmol m^-2^ s^-1^, 10 min). Fast phase (0–250 ms after switching off the actinic light) of the dark-interval relaxation kinetics was fitted with a single exponential decay function. The reciprocal value of the halftime of the electrochromic shift (ECS) decay, i.e., the thylakoid conductivity (gH^+^), was used as a measure of ATP synthase activity because fast ECS decay is exclusively attributable to proton efflux through the ATP synthase (Avenson et al., 2005).

### Measurements of Gas Exchange Parameters

Gas exchange parameters were measured using LI-6400 (Li-COR, Inc., Lincoln, NE, United States) according to the manufacturer’s instruction. Measurements were taken between 10:00 to 14:00 daily and data acquisition was done as previously (Li et al., 2016). Determinations of gas exchange parameters were made on at least three leaves from different seedlings of all replicates. Data were recorded and analyzed with data acquisition software (OPEN 5.3, LI-COR).

## Results

### Impact of Pi-Deprivation on Shoot Growth of *L. chinensis*

**FIGURE 1:**
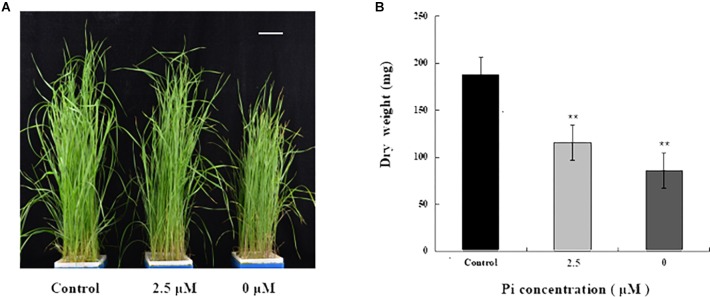
Growth and biomass accumulation in above-ground tissues of *L. chinensis* grown under different Pi concentrations. **(A)** A representative photograph of seedlings grown under Pi-repleted (250 μM, control), Pi-deprived (0 μM) and low-Pi (2.5 μM) conditions for 4 weeks. Scale bar represents 5 cm. **(B)** Biomass accumulation in the above-ground tissues of the seedlings grown at the indicated Pi concentrations. Experiments were repeated three times with similar results. The data shown are from one of the experiments with three replicates. Error bars represent standard deviation of the mean (*n* = 15). ^∗∗^Refers to *p*-values <0.01 in Student’s *t*-test.

To investigate the impact of Pi-deprivation stress on *L. chinensis*, we initially compared the growth of the seedlings grown under Pi-repleted (250 μM) and Pi-deprived (0 μM) conditions, respectively. Since the low-Pi stress treatment (2.5 μM) is known to be effective in triggering Pi-starvation responses in *L. chinensis* (Li et al., 2016), this stress treatment was also included for comparison. Germinated seeds of *L. chinensis* were hydroponically grown under Pi-repleted (250 μM) condition for 4 weeks and then transferred to Pi-starved (0, 2.5 μM) as well as the control conditions with normal Pi supply (250 μM) for 4 weeks, respectively. Figure 1A shows that shoot growth of *L. chinensis* under Pi-deprived condition was largely repressed compared to the control (250 μM) as well as to the low-Pi (2.5 μM) treated plants. To quantify the impact of Pi-deprivation on above-ground tissues, biomass accumulated in the shoots was determined. Figure 1B presents the average data from three replicates consisting of fifteen seedlings. Compared to control, biomass of the Pi-deprived and low-Pi seedlings decreased 55.3 and 36.8%, respectively, after 4-week of stress treatments (Figure 1B).

### Reduced Pi Contents in *L. chinensis* Under Pi-Deprivation

**FIGURE 2:**
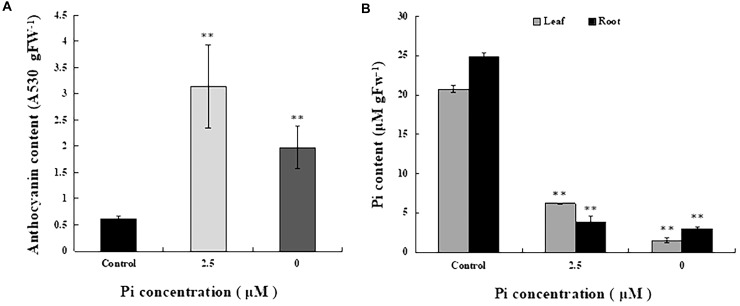
Anthocyanin and Pi contents in seedlings grown under different Pi concentrations. **(A)** Anthocyanin contents of the third leaves from control and Pi-deficient seedlings. **(B)** Pi content in leaf and root tissues of seedlings grown under different Pi concentrations. Experiments were repeated three times with similar results. The data shown are from one of the experiments with three replicates. Error bars represent standard deviation of the mean (*n* = 15). ^∗∗^Refers to *p*-values <0.01 in Student’s *t*-test.

To determine the extent of Pi deficiency in *L. chinensis* caused by Pi-deprived stress treatment, we measured anthocyanin and Pi contents in the seedlings. Figure 2A shows that the level of anthocyanin in the leaves of Pi-deprived seedlings, as expected, was substantially higher than that of control plants. Dramatic decline of Pi contents in both roots and leaves of the Pi-deprived seedlings was also observed (Figure 2B). After 4-week of Pi-deprivation, only 10% (in roots) and 15% (in leaves) of the control level was remained, leading to severe Pi deficiency in *L. chinensis*. Our measurements also revealed that, in contrast to the low-Pi stressed seedlings, Pi content of roots was clearly higher than that of leaves, resulting in the ratio R/L > 1 in the Pi-deprived plants (Figure 2B). The distinct Pi allocation patterns and anthocyanin levels between Pi-deprived (0 μM) and low-Pi (2.5 μM)-stressed *L. chinensis* (Figure 2) raise the possibility that Pi-deficiency responses caused by Pi-deprivation could be different from that induced by low-Pi stress.

### Reduced PSII Activity in Pi-Deprived *L. chinensis*

To test the possibility mentioned above, we first compared photochemical activity of the *L. chinensis* plants under different Pi supplied conditions. Photochemical activity of the *L. chinensis* plants was assessed based on *in vivo* chlorophyll *a* fluorescence measurements with a Dual PAM-100 chlorophyll fluorimeter (Heinz Walz, Germany) (Table 1). While maximal photo-oxidizable P_700_ (P_700_) was similar, Fv/Fm (maximum quantum yield) and φ(II) (effective quantum yield) of the Pi-deprived plants decreased 15.1 and 29.3%, respectively, relative to the control plants. These indicate that PSII activity was significantly affected due to Pi-deprivation. The measurements also show that NPQ (non-photochemical quenching) in Pi-deprived plants was enhanced (2.7-fold higher than the control), implicating that the thermal dissipation of excitation energy in PSII was increased under such stress condition. Comparison of the values, however, revealed no difference in the chlorophyll fluorescence parameters and chlorophyll contents between Pi-deprived and low-Pi-stressed seedlings (Table 1).

**Table 1:** Chlorophyll fluorescence parameters of leaves from control and Pi-deficient seedlings.

Treatment	Chl content (mg g Fw^-1^)	Fv/Fm	Φ(II)	NPQ	P_700_
Control	3.30 ± 0.20	0.73 ± 0.02	0.58 ± 0.06	0.35 ± 0.11	1.10 ± 0.00
2.5 μM	3.08 ± 0.08	0.62 ± 0.02^∗∗^	0.40 ± 0.05^∗∗^	0.99 ± 0.17^∗∗^	1.08 ± 0.00
0 μM	3.06 ± 0.27	0.62 ± 0.02^∗∗^	0.41 ± 0.06^∗∗^	0.93 ± 0.19^∗∗^	1.09 ± 0.00


*Fv/Fm and Φ(II) represent the maximal and effective photochemical quantum yield of PSII, respectively. *NPQ* is non-photochemical quenching calculated as (Fm-Fm’)/Fm’. P_700_ is the estimation of PSI photochemical activity. Mean values ± SD (*n* = 9) are provided. ^∗∗^Refers to *p*-values <0.01 in Student’s *t*-test.*

**FIGURE 3:**
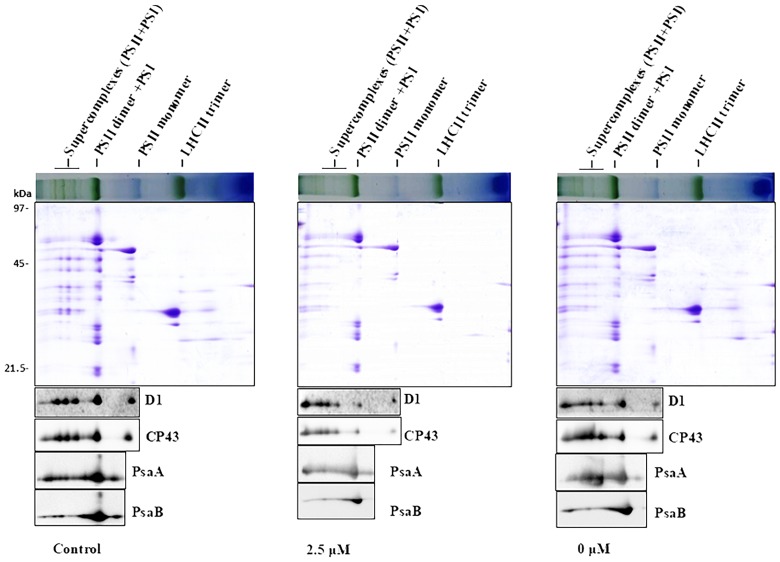
2-D BN/SDS-PAGE analysis of thylakoid membrane complexes from the leaves of control and Pi-stress-treated seedlings. Membranes (equivalent to 5 μg chlorophyll) solubilized with 1% n-dodecyl-β-D-maltoside were separated by BN-PAGE in the first dimension (upper panel) followed by separation of the proteins in the second dimension using 15% SDS/PAGE (middle panel) and subsequent immunoblot detection with antibodies specific to subunits of PSII (α-D1, α-CP43) and PSI (α-PsaA, α-PsaB), respectively. Photosynthetic membrane complexes were designated according to (Peng et al., 2008; Chen et al., 2013). Similar results were obtained in two additional independent experiments.

To obtain further insights of Pi-deprivation effect on photosynthetic apparatus, we analyzed thylakoid membrane complexes of *L. chinensis* by 2-D blue native/SDS-PAGE (2D-BN/SDS-PAGE) combined with immunoblot analysis using the published protocol (Peng et al., 2008; Yang et al., 2014; Zhao et al., 2017). Thylakoid membranes prepared from three independent experiments were solubilized with dodecyl-β-D-maltopyranoside (DM; 1%) followed by separation using BN-PAGE in the first dimension (Supplementary Figure S1A; top panels of Supplementary Figure S1B; top panels of Figure 3). The three gels (with three biological replicates) showed similar visual patterns and revealed that, unexpectedly, the reduction of the complexes in the Pi-deprived seedlings (0 μM) was less pronounced than that under low-Pi stress condition. The protein subunits were then separated in the second dimension (15% SDS-PAGE) followed by immunoblotting detection with the antibodies against core subunits of both photosystems (D1, CP43, PsaA, PsaB) (Figure 3 and Supplementary Figure S1B). SDS-PAGE gel pattern and immunoblotting detection was highly similar between the biological replicates showing the intermediate levels in Pi-deprived samples (between control and low-Pi-stressed seedlings). Based on these experimental data, we conclude that the structure of thylakoid protein complexes in Pi-deprived seedlings is slightly more stable than those under low-Pi-stressed condition.

### Changes in MDA Content and Anti-ROS Activity Under Pi-Deprivation

**FIGURE 4:**
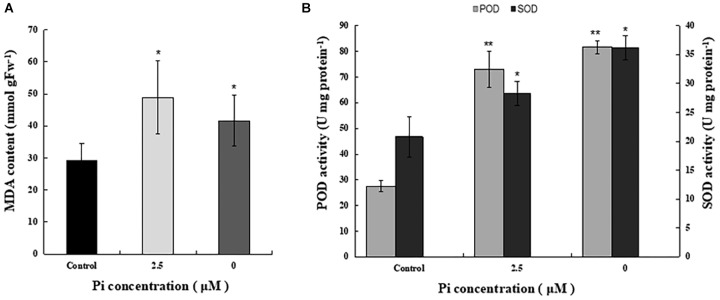
Comparison of MDA content and activity of ROS-scavenging enzymes in the third leaves of control and Pi-stress-treated seedlings. **(A)** MDA content determined via the thiobarbituric acid reactive substance assay. **(B)** Peroxidase (POD) and superoxide dismutase (SOD) activity. The experiment was repeated three times with similar results. The data shown are from one of the experiments with three replicates. Error bars represent standard deviation of the mean (*n* = 9). ^∗^, ^∗∗^Refer to *p*-values <0.05 and <0.01 in Student’s *t*-test, respectively.

To examine the impact of Pi-deprivation on photosynthetic membrane lipids, we estimated the extent of lipid peroxidation by quantification of MDA in the leaves of the plants under different Pi levels (Figure 4A). Compared to the control, the level of MDA increased 29.3% in the Pi-deprived seedlings. This is significantly lower than that of low-Pi-stressed plants (Figure 4A), suggesting that lipid peroxidation of their thylakoid membranes was less than those under low-Pi stress condition. Activity measurements of several reactive oxygen species (ROS) scavenger enzymes, i.e., superoxide dismutases (SOD) and peroxidases (POD), in the leaves of the corresponding plants also show the highest values in the Pi-deprived seedlings (Figure 4B). Together with the slightly lower NPQ (Table 1), we conclude that in the duration of Pi-stress treatments, photosynthetic membranes of Pi-deprived *L. chinensis* was slightly better protected compared to the low-Pi (2.5 μM)-stressed seedlings.

### Decreased ATP Synthase Activity Under Pi-Deprivation

**FIGURE 5:**
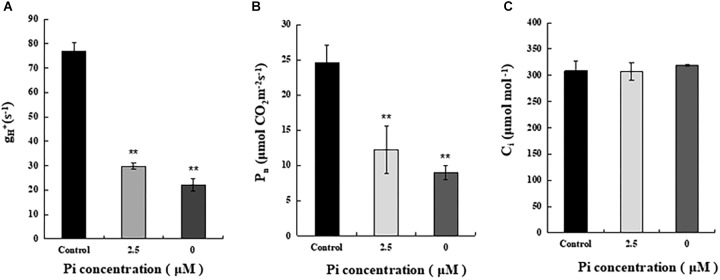
Measurements of ATP synthase activity and net photosynthesis rate (Pn) of the third leaves from control and Pi-stress-treated seedlings. **(A)** ATP synthase activity was estimated by thylakoid membrane H^+^ conductivity (gH^+^) calculated as gH^+^ = 1/τ, where τ is the time constant for decay determined by fitting a single exponential decay function to the electrochromic shift (ECS) decay signal obtained after the actinic light (754 μmol m^-2^ s^-1^) was switched off. Standard deviations were estimated from three biological replicates. Experiments were repeated twice and similar results were obtained. **(B,C)** Net photosynthesis rate (Pn) and intercellular CO_2_ concentration (Ci) of the leaves from control and Pi-stress-treated seedlings, respectively. Experiments were repeated three times with similar results. The data shown are from one of the experiments with three replicates. Error bars represent standard deviation of the mean (*n* = 15). ^∗∗^Refers to *p*-values <0.01 in Student’s *t*-test.

To understand why reduction of shoot growth and biomass accumulation was more pronounced in Pi-deprived seedlings than that under low-Pi stress condition (Figure 1), we then compared ATP synthase activity of control, Pi-deprived (0 μM) and low-Pi (2.5 μM)-stressed *L. chinensis* (Figure 5A). The enzyme activity was estimated by measuring the H^+^ conductivity through ATP synthase (gH^+^) using the Dual PAM-100 instrument equipped with a P515/535 module (Heinz Walz, Effeltrich, Germany) (Schreiber and Klughammer, 2008). Measurements were performed by following the manufacturer’s instructions and as described (Duan et al., 2016; Zhang et al., 2018). Our experimental data shows that, compared to the control, ATP synthase activity of Pi-deprived seedlings decreased 71.4%. This was 10.4% lower than that under low-Pi stress (Figure 5).

Because ATP produced by ATP synthase is essential for CO_2_ assimilation in chloroplasts, it is possible that the significantly decreased ATP synthase activity described above may cause greater reduction of photosynthetic efficiency in the Pi-deprived seedlings. To verify this, we measured gas exchange parameters of the third leaves from each treatment using an open gas exchange system (Li-6400; Li-COR, United States) by following the manufacturer’s instruction. The data shows that the net photosynthetic rate (Pn) of Pi-deprived plants was the lowest and exhibited 36.7% of the control level, which was 12.3% lower than low-Pi stressed seedlings (Figure 5B). Since no difference in the intercellular CO_2_ concentrations (Ci) was observed between control and Pi-stressed plants (Figure 5C), and the reduced portion of ATP synthase activity was largely comparable to the decreased Pn between Pi-deprived and low-Pi-stressed plants (Figure 5), we suggest that the greater growth/biomass reduction of Pi-deprived *L. chinensis* seedlings could be largely attributable to the reduced capability of ATP production catalyzed by ATP synthase in the thylakoids.

## Discussion

In this study, we have investigated growth and photosynthetic characteristics of the dominant forage species *L. chinensis* in response to Pi-deprivation. We show that the shoot growth inhibition was most obvious in the Pi-deprived seedlings. We also show the lower biomass accumulation and net photosynthesis rate (Pn) in the Pi-deprived seedlings than that under low-Pi-stressed condition. We found that the reduction of ATP synthase activity of Pi-deprived seedlings was significantly greater and in accordance with the decreased Pn under such stress condition. New insightful findings obtained from the present work are discussed as below.

Pi-supply is one of the major management practices toward sustaining plant productivity (Cordell et al., 2009; MacDonald et al., 2011). To minimize Pi application in the nutrient medium of cultivation systems, knowledge of the tolerance of economically important plants to Pi deficiency as well as their physiological basis is essential. In this research work, we investigated Pi-deficiency resistance of *L. chinensis* via transferring the seedlings into a medium lacking Pi (Pi-deprivation). We observed remarkable inhibition of shoot growth of *L. chinensis*. This is in agreement with earlier observations in numerous plant species including Arabidopsis and barley and (Mimura et al., 1996; Rouached et al., 2011; Veneklaas et al., 2012). Also, we found that the Pi-deprivation treatment (-Pi for 4 weeks) caused 55.3% biomass reduction in the above-tissues of *L. chinensis* (Figure 1). Based on our data from the parallel experiments, we estimate that this biomass reduction was 18.5% more than that under low-Pi (2.5 μM) stress condition. These are the valuable experimental information regarding Pi-supply for the important forage species.

Photosynthesis is the basis of biomass accumulation in plants. While remarkable inhibition of photosynthesis by Pi-deprivation was largely expected, we found no difference for PSI as well as PSII in terms of photochemical activity between the Pi-deprived and low-Pi-stressed *L. chinensis* (Table 1 and Figure 3). This suggests that the greater reduction of biomass accumulation in Pi-deprived plants is largely due to impairment of other components in the photosynthetic machinery. Our experimental data showing that the decline of the ATP synthase activity, which was revealed from the measurements of proton conductivity through ATP synthase (gH^+^), of the Pi-deprived seedlings was significantly greater than that under low-Pi-stressed condition (Figure 5), is of strong support for the assumption. Because the reduced ATP synthase activity is correlated exclusively with the decreased Pn (Figure 5), we suggest that the greater decrease of photosynthetic efficiency (Pn) in the Pi-deprived seedlings is mainly due to the reduced capability of ATP production catalyzed by ATP synthase in the thylakoids of *L. chinensis*.

It is intriguing to understand how ATP synthase activity was regulated in *L. chinensis* under Pi deficiency. Clearly, Pi concentration in the leaves from Pi-deprived seedlings was slightly lower than that of low-Pi-stressed plants (Figure 2). It is possible that the difference in ATP synthase activity described above is due to fine-tuning of the enzyme activity. Though direct experimental evidence is lacking, it has been earlier speculated that the fine-tuning might operate by monitoring metabolites including Pi needed for ATP synthesis in chloroplasts (Avenson et al., 2005; Takizawa et al., 2008).

It is interesting to note that thylakoid membrane protein complexes of Pi-deprived seedlings were more stable than that under low-Pi-stressed condition (Figure 3). This is of indication that, in the duration of Pi-stress treatments, photosynthetic membranes of Pi-deprived seedlings was somehow better protected than that under low-Pi stress. Our findings of decreased lipid peroxidation and increased anti-ROS activity in the Pi-deprived seedlings relative to the low-Pi stressed samples (Figure 4) is compatible with this assumption. Further elucidation of the sensing and photoprotection mechanisms under low-Pi and Pi-deprivation conditions will gain more insights of the signaling pathways in the organism.

In summary, our present work revealed growth and photosynthetic characteristics of *L. chinensis* in response to Pi-deprived stress. We found rapid inhibition of shoot growth and significant biomass reduction as well as remarkable decrease of photosynthetic efficiency in the leaves of Pi-deprived seedlings. We also compared the responses with the seedlings subjected to low-Pi stress condition. We found that the reduced shoot biomass accumulation in the Pi-deprived plants relative to the low-Pi-stressed plants could be largely correlated with the decreased ATP synthase activity. We hypothesize that the decreased growth/biomass accumulation in the Pi-deprived seedlings relative to the low-Pi stressed plants is mainly attributable to the reduced capability of ATP production catalyzed by ATP synthase in the thylakoids of *L. chinensis*.

## Author Contributions

FH, XW, and LP conceived the research and designed the experiments. LL, HY, and PL performed the experiments. WR and JG contributed to materials. LL and FH analyzed the data and wrote the manuscript.

## Conflict of Interest Statement

The authors declare that the research was conducted in the absence of any commercial or financial relationships that could be construed as a potential conflict of interest.

## Supplementary Material

FIGURE S1 Thylakoid membrane complexes of two additional biological replicates separated by BN-PAGE **(A)** and 2D-BN/SDS-PAGE **(B)**. Experimental details were same as described in the legend of Figure 3.
