# Supplementary figures and images for: Comparative Study Reveals Insights of Sheepgrass (Leymus chinensis) Coping With Phosphate-Deprived Stress Condition
Source: Front Plant Sci. 2019 Feb 19;10:170. doi: 10.3389/fpls.2019.00170 (PMC6401631; doi:10.3389/fpls.2019.00170)

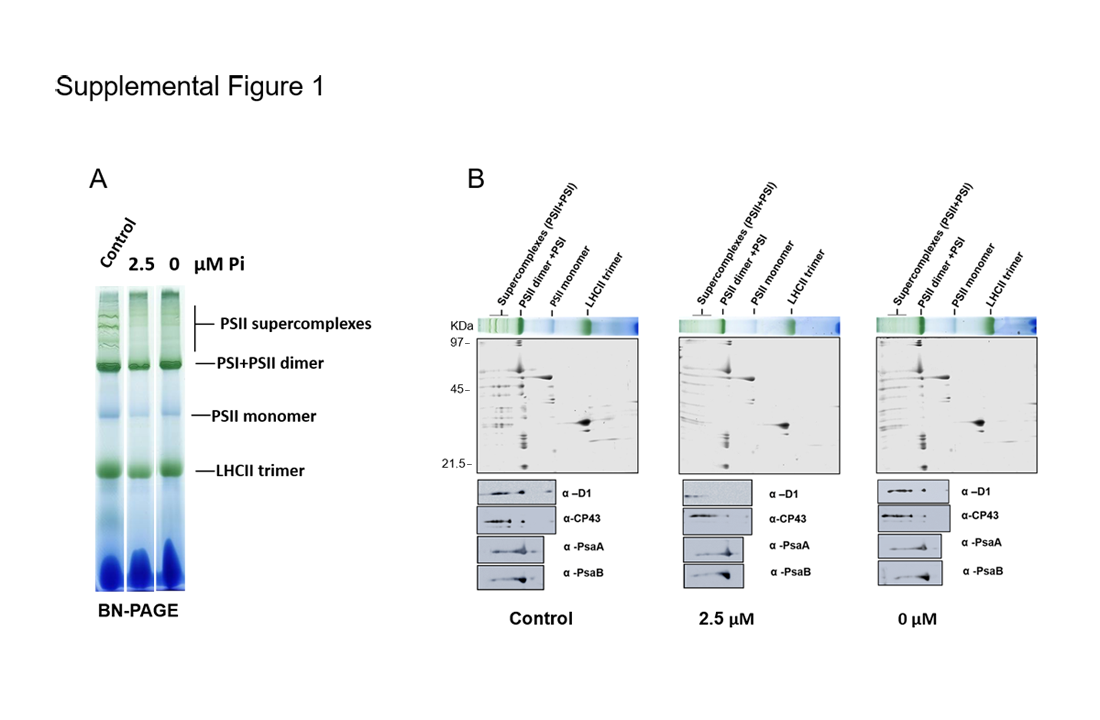

Supplement: FIGURE S1 — Thylakoid membrane complexes of two additional biological replicates separated by BN-PAGE (A) and 2D-BN/SDS-PAGE (B). Experimental details were same as described in the legend of Figure 3. [file Image_1.TIF]
